# Defining the dance: quantification and classification of endoplasmic reticulum dynamics

**DOI:** 10.1093/jxb/erz543

**Published:** 2019-12-07

**Authors:** Charlotte Pain, Verena Kriechbaumer

**Affiliations:** Oxford Brookes University, Faculty of Health and Life Sciences, Gipsy Lane, Plant Cell Biology, Oxford, UK

**Keywords:** Dynamics, endoplasmic reticulum (ER), ER remodeling, microscopy, movement, quantitative analysis

**The availability of quantification methods for subcellular organelle dynamic analysis has increased rapidly over the last 20 years. The application of these techniques to contiguous subcellular structures that exhibit dynamic remodelling over a range of scales and orientations is challenging, as quantification of ‘movement’ rarely corresponds to traditional, qualitative classifications of types of organelle movement. The plant endoplasmic reticulum represents a particular challenge for dynamic quantification as it itself is an entirely contiguous organelle that is in a constant state of flux and gross remodelling, controlled by the actinomyosin cytoskeleton.**

## History and why it matters

**Fig. 1.**
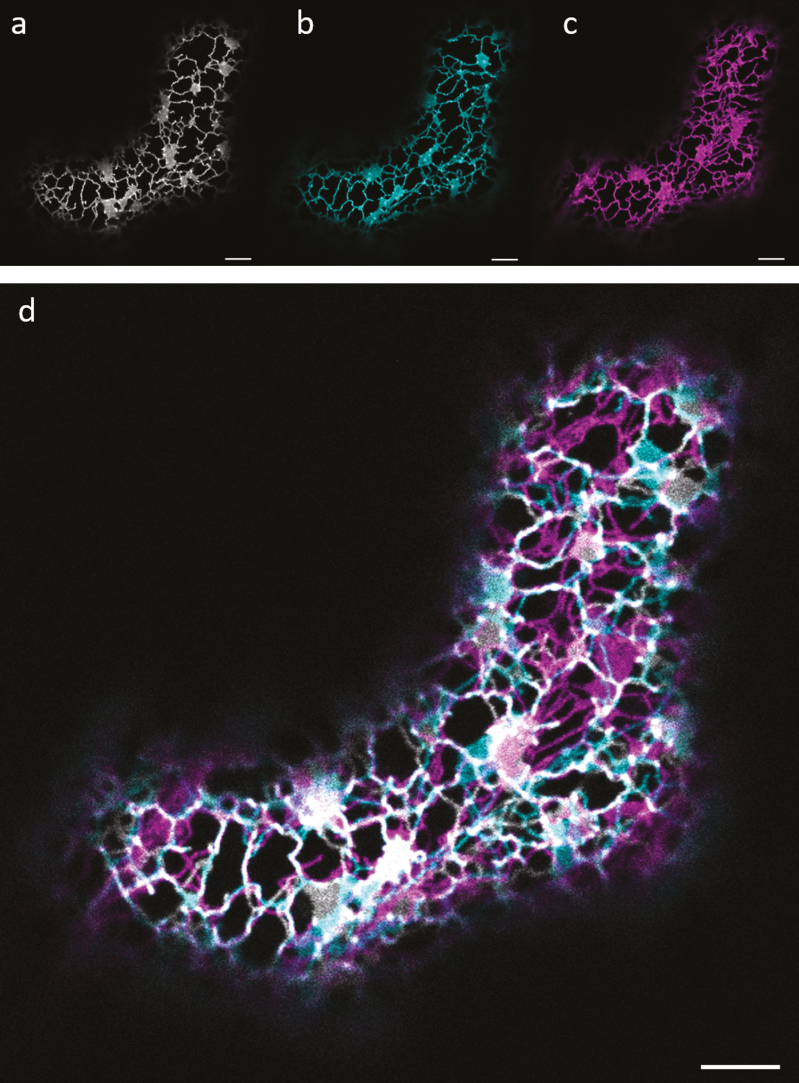
Temporal colour coding to represent ER remodelling. (a–c) Single frames of a tobacco leaf epidermal cell transiently expressing the ER marker GFP–HDEL. Images are collected 60 s apart and are individually pseudocoloured. (d) Overlay of all pseudocoloured frames to produce a temporal colour-coded image of ER dynamics through time. Scale bars=5 µm.

The dynamics of the endoplasmic reticulum (ER) are complex and occur over a range of scales. The most commonly described form of ER movement is the gross structural remodelling first identified by Ridge *et al,* (1999) with the development of live-cell fluorescence imaging techniques. The ER undergoes rapid and extensive remodelling around a number of fixed nodes (Lin *et al.*, 2014). This movement is largely abolished in the absence of a functional actinomyosin cytoskeleton (Ueda *et al.*, 2010; Griffing *et al.*, 2014; Hawes *et al.*, 2015; Pain *et al.*, 2019), though some Brownian/disorganized motion of ER junctions can be detected after pharmacological treatment with latrunculin B (Lat B), preventing polymerization of the actin cytoskeleton (Lin *et al.*, 2017; Pain *et al.*, 2019). This remodelling of the ER network is often displayed as simple image overlays to provide a temporal colour coding of the shifting ER network (Ridge *et al.*, 1999; Cao *et al.*, 2016; Griffing *et al.*, 2017; Kriechbaumer *et al.*, 2018) (Fig. 1). Though providing a visual overview of ER remodelling, it does not provide any quantitative information on the rate of ER remodelling.

In recent years, numerous computational methods have been developed in conjunction with improved live-cell imaging to quantify and analyse the rate at which the ER remodels. These methods are useful but often do not directly correlate to previous descriptions of ER movement, instead characterizing the movement of the ER network as a total entity rather than analysing the movement of its constituent parts. With the development of fluorescence recovery after photobleaching (FRAP), single molecule tracking, and photoactivatable and photoconvertible fluorophores, the movement of the ER at a molecular level can now be interrogated (Runions *et al.*, 2006; Sparkes *et al.*, 2008, 2009; Tolley *et al*., 2008, 2010; Mathur *et al.*, 2010; Griffing *et al.*, 2014; Breeze *et al.*, 2016; Holcman *et al.*, 2018). This movement is necessarily conflated with the general remodelling of the ER, yet potentially offers unique insights into protein function on the surface of the ER.

**Fig. 2.**
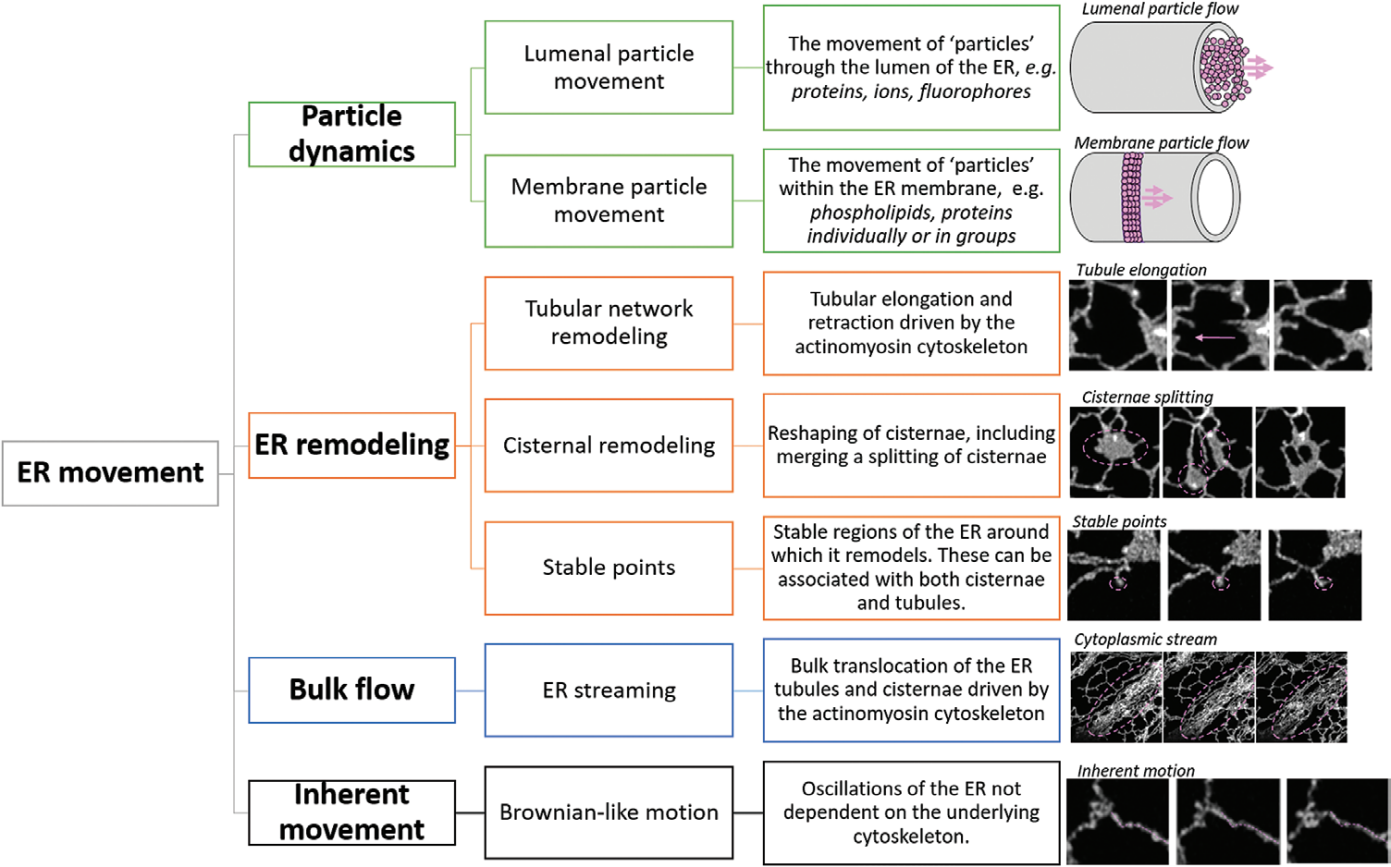
Proposed classification of types of ER dynamics. Four proposed classifications of ER dynamics split over a range of scales and across different structures. Examples of several types of movement, including schematic representations of lumenal versus membrane particle flow, tubule elongation, cisternae splitting, the identification of stable nodes, rapid ER streaming, and inherent ER motion after treatment with Lat B are also shown. All images are of tobacco leaf epidermal cells transiently expressing GFP–HDEL. Images are captured using confocal microscopy.

Here we propose a classification of ER movement that correlated more directly with the methodology used to characterize ER movement whereby we divide ER movement into four classes, each applying over a different scale (Fig. 2): (i) ER particle dynamics; (ii) ER remodelling; (iii) bulk flow; and (iv) inherent ER movement.

## Endoplasmic reticulum particle dynamics

**Fig. 3.**
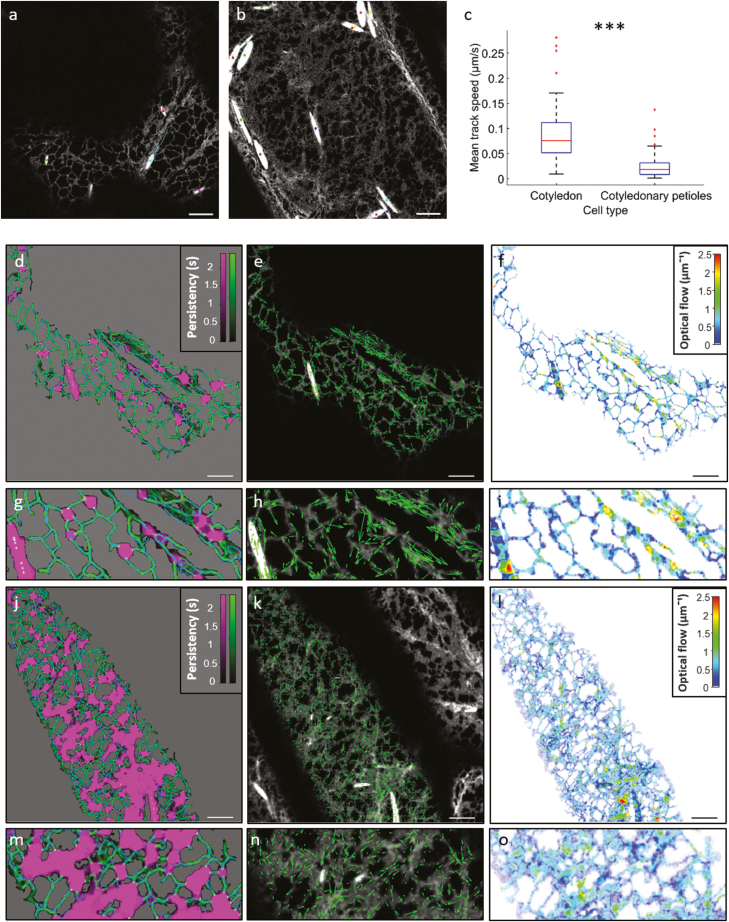
Detection of ER lumenal particle dynamics and ER remodelling in two Arabidopsis cell types. TrackMate output of the path of Arabidopsis fusiform body movement through the lumen of the ER in Arabidopsis 7-day-old cotyledon cells (a) and cotyledonary petiole cells (b). Boxplot of the mean speed of fusiform body movement in the two cell types (c). Persistency mapping (d), the direction of ER remodelling detected by optical flow (e), and the rate of ER remodelling detected using optical flow (f) in cotyledon cells, with enlarged regions shown below (g–i). Persistency mapping (j) and optical flow analysis outputs (k and l) of ER remodelling analysis in Arabidopsis cotyledonary petioles, with enlarged regions shown below (m–o). Persistency maps are displayed for cisternae (magenta), tubules (green), and stable points (white), with the skeleton from the initial frame overlaid in blue. Darker colours indicate less stable structures. Optical flow images are pseudocoloured by the rate of detected movement between frames. Scale bars=5 µm.

ER particle dynamics refers to the movement of any particle, or group of particles, capable of moving through either the lumen or membrane of the ER. Particle is a general term, referring to individual proteins, protein complex, lipids, ions, and whole, contiguous structures such as Arabidopsis fusiform bodies (Hawes *et al.*, 2001). Quantification of the dynamics of large particles such as Arabidopsis fusiform bodies through the lumen of the ER is relatively trivial. An analysis using tracking software such as TrackMate (Tinevez *et al.*, 2017) reveals that the movement of such large particles is significantly reduced in Arabidopsis cotyledonary petioles compared with cotyledonary cells (Fig. 3a–c, *P*-value 2.12×10^–5^). The reduction of particle movement in this case also corresponds to a reduction in the rate of tubule remodelling in cotyledonary petiole cells (Fig. 3d–o), suggesting a potential link between ER remodelling and particle dynamics within the lumen of the ER.

Lumenal particle dynamics might also consider the movement of the ER lumen-resident proteins such as calreticulin (Opas *et al.*, 1991). The movement of lumenal particles can be affected by ER tubule architecture (Holcman *et al.*, 2018) and by the lumenal space through which they can move. Plant ER tubules have an estimated width of 40 nm (Pain *et al.*, 2019), but this width can be reduced extensively by overexpression of members of the reticulon protein family which can cause tubular constriction to such as extent that lumenal particle movement is restricted to subdomains of the ER (Tolley *et al.*, 2008; Sparkes *et al.*, 2010; Breeze *et al.*, 2016). The movement of particles within the membrane of the ER is distinct from that of particles within the ER lumen as the proteins or protein complexes on the ER membrane may be affected by binding to cellular components outside the ER. For example, NET3C punctae associated with the surface of the ER (Deeks *et al.*, 2012; Wang *et al.*, 2014) show increased recovery from photobleaching after the depolymerization of the actin cytoskeleton by Lat B (Wang *et al.*, 2014), despite Lat B being known to reduce the rate of ER remodelling (Sparkes *et al.*, 2009; Pain *et al.*, 2019). The composition of the membrane domains surrounding particles of interest may also affect the way in which particles move through the ER membrane. Though not yet identified in plant ER, solid-phase domains of mammalian ER associated with lipid biosynthesis have been identified (Shen *et al.*, 2017). Incorporation of particles into these domains is likely to affect motility within the ER membrane. Furthermore, ER lumen-resident cargo molecules such as vesicular stomatitis virus glycoprotein (VSV-G) selectively interact with the Sar1^GTP^·Sec23/24 ternary complex as part of selective cargo sorting prior to packaging into COPII vesicles (Aridor and Traub, 2002; Aridor *et al.*, 1998). During this period of interaction, the movement of soluble cargo proteins through the ER lumen is likely to be affected by the movement of Sar1^GTP^·Sec23/24 within the ER membrane.

Methods available to assess particle dynamics in the plant ER are typically fluorophore bleaching/photoactivation experiments focusing on the recovery of a bleached fluorophore to a specific region of the ER, or tracking the spread of a photo-activated fluorophore through the ER network (Sparkes *et al*., 2006, 2009; Griffing, 2018). Lumenal and membrane particle flow can also be analysed using single particle tracking of fluorophores using variable-angle epifluorescence microscopy (VAEM), so far applied near the surface of the cell (Konopka and Bednarek, 2008), but can also be used to detect particles at a sufficient depth in the cell to capture ER particle dynamics in mammalian cells (Holcman *et al.*, 2018). The challenge of using imaging techniques is separating the movement of particles within or on the surface of the plant ER from gross ER remodelling.

## Endoplasmic reticulum remodelling

ER remodelling refers to the gross restructuring of the ER driven by the actinomyosin cytoskeleton. This remodelling usually occurs at a rate of 0.3 µm s^–1^ (Ueda *et al.*, 2010). Typically, descriptions of the remodelling processes are divided by the morphological subregions involved. Tubules are described as remodelling through extension, retraction, and sliding of individual tubules over variable time periods, whilst cisternae remodel though merging, splitting, translocation through the ER, and general reshaping of the cisternae. Currently only tubule remodelling can be analysed at a relatively small scale of a few tubules per time series, where each tubule can be reduced to single pixel-wide skeletons along the ridge of ER tubules for active ER dynamic analysis (Lemarchand *et al.*, 2014; Lin *et al*., 2014, 2015, 2017). As yet this software cannot be applied to cisternae and has not yet been applied to whole cells for analysis. For whole-cell quantification of ER remodelling, two key methods have been employed thus far: optical flow analysis and persistency analysis.

Optical flow and other cross-correlation methods capture the apparent movement of objects between frames across the entire image. Numerous methods for cross-correlation analysis are available, including KbiFlow (Ueda *et al.*, 2010) and AnalyzER (Pain *et al.*, 2019). Though these methods provide a measurement for ER movement across the cell, unlike the methods applied by Lin *et al*. (2014, 2015, 2017) and Lemarchand (2014), they are not able to identify the types of movement occurring, only how fast regions of the ER appear to be moving. A second component of ER remodelling are the sections of the ER that are not in fact moving. Though stable points of the ER, which are ‘persistent’ and therefore not remodelling, will be identified by cross-correlation analysis methods, the length of time over which they remain persistent is not calculated using cross-correlation. Persistency is typically calculated by measuring pixel occupation through time, that is for how long a region of the ER has remained included within a single pixel and has been used to assess the role of myosin in remodelling (Sparkes *et al.*, 2009; Griffing, 2010; Griffing *et al.*, 2014; Pain *et al.*, 2019).

## Bulk flow

Bulk flow refers to the rapid, mass translocation of regions of ER driven by the actin cytoskeleton in concert with myosin motors, in particular myosin XI-K (Ueda *et al.*, 2010). This form of ER movement is distinct from ER remodelling as it is significantly more rapid, occurring at speeds of up to 1.35 µm s^–1^ compared with remodelling that is thought to occur at a rate of ~0.3 µm s^–1^ (Ueda *et al.*, 2010). In addition, ER streaming forms a distinct structure that can be identified from still images. Streaming ER forms long strands of densely grouped, highly reticulate sections of ER, as opposed to non-streaming ER which forms a significantly less dense structure. Due to the density of the ER network within these streams, persistency analysis and single tubule analysis methods struggle to characterize ER movement. Measuring bulk flow would currently typically require cross-correlation methods such as optical flow (Ueda *et al.*, 2010).

## Inherent endoplasmic reticulum movement

Inherent ER movement is the small oscillatory movement of the ER that continues in the absence of known ER remodelling drivers. For example, constant, small oscillations of relatively stable points of ER continue after depolymerization of the actin cytoskeleton using drug treatments such as Lat B. This small oscillation can be observed at the edge of cisternae as well as along tubules, and contributes to the overall movement of the ER, though it is only observable when rapidly imaging the ER and in the absence of larger ER movement (Lin *et al.*, 2017; Pain *et al.*, 2019).

## Conclusion

Proposed here is an ER dynamics classification system, developed in the context of currently available image analysis techniques used for dynamic quantification. The ultimate goal of ER dynamic analysis is to track the movement of single particles through the ER or, on a large scale, perform ‘single feature tracking’ such as following the fate of individual morphological domains of the ER, such as cisternae, tubules, and three-way junctions. This is currently hindered by both available imaging technology and image analysis methods. The ER not only rapidly remodels, but also fluidly interconverts between different structure domains whilst remodelling. For example, cisternae identified in at an initial time frame can rapidly merge with other tubules and three-way junctions or may collapse into these various components. To capture these events and accurately track the changing morphological features will require improved time resolution on widely available microscopes, but also increasingly computationally expensive methods, such as those applied by Lin *et al.* (2014) and Pain *et al.* (2019), but on a much larger scale. Additional resolution in time may have the added benefit of allowing future work into teasing apart the structure of the ER undergoing bulk flow. With the current developments of single particle tracking at the plasma membrane of plant cells (Wan *et al.*, 2011; Martiniere *et al*., 2012; Hutten *et al.*, 2017; Chen and Tian, 2018; McKenna *et al.*, 2019) and within the mammalian ER (Holcman *et al.*, 2018), single particle tracking through the ER will be achievable. The different types of ER movement are strongly interlinked and will influence movement of single particles and features, but–for example—separation of such single particle movement from ‘background’ ER remodelling or membrane flow will be one of the greater future challenges in understanding ER functioning.
